# Diagnostic-Therapeutic Pathway and Outcomes of Early Stage NSCLC: a Focus on EGFR Testing in the Real-World

**DOI:** 10.3389/fonc.2022.909064

**Published:** 2022-06-29

**Authors:** Giulia Pasello, Martina Lorenzi, Giulia Pretelli, Giovanni Maria Comacchio, Federica Pezzuto, Marco Schiavon, Alessandra Buja, Stefano Frega, Laura Bonanno, Valentina Guarneri, Fiorella Calabrese, Federico Rea

**Affiliations:** ^1^Department of Surgery, Oncology and Gastroenterology, University of Padua, Padua, Italy; ^2^Division of Medical Oncology 2, Veneto Institute of Oncology IOV – Istituto di Ricovero e Cura a Carattere Scientifico (IRCCS), Padua, Italy; ^3^Thoracic Surgery Unit, Department of Cardio-Thoracic and Vascular Sciences, University of Padua, Padua, Italy; ^4^Pathology Unit, Department of Cardio-Thoracic and Vascular Sciences, University of Padua, Padua, Italy; ^5^Department of Cardiologic, Vascular, and Thoracic Sciences and Public Health, University of Padua, Padua, Italy

**Keywords:** NSCLC, early-stage, real-world, EGFR, costs

## Abstract

**Background:**

Osimertinib is considered the standard-of-care for previously-untreated *EGFR* mutant advanced non-small cell lung cancer (NSCLC). Oncogene driver screening in early NSCLC is not standard practice. A real-world study has been designed in order to investigate the optimal testing frequency and timing for *EGFR* mutations in early NSCLC in clinical practice.

**Patients and Methods:**

The present observational, retrospective study evaluated the real-world diagnostic-therapeutic pathway and clinical outcomes of 225 patients with stage I-III NSCLC, with particular reference to the *EGFR*-mutant subgroup.

**Results:**

Prior to surgery, 101 patients had undergone a diagnostic biopsy; *EGFR* mutational analysis was available in 56 (55%) patients and 12 patients (21%) had a cancer harboring an *EGFR* mutation. Among surgical specimens, reflex *EGFR* test was performed in 181 (80%) of 225 and 35 cases (19%) were *EGFR* mutant. The majority of patients had not received adjuvant chemotherapy (*N*=174, 77%) or adjuvant radiotherapy (N=201, 89%). Of 49 (22%) patients experiencing disease relapse, 26 (53%) received first-line systemic treatment. All *EGFR*-mutant relapsed patients (N=6, 12.2%) received an EGFR-TKI. Median overall survival (OS) and relapse-free survival for the entire population were not reached. Multivariate analysis for OS confirmed a significant correlation with age, female gender, *EGFR* status, necrosis score, perineural invasion, and relapsed disease. *EGFR* test costs represented 1.6-2.4% of the total costs of management per patient (€34,340).

**Conclusions:**

Our results suggest that the frequency of *EGFR* mutations in early stage (I-III) NSCLC is similar to that of advanced stages. Reflex *EGFR* testing in all early-stage NSCLC at diagnosis or after surgery appears to be a valid tool to give patients the chance to benefit from targeted adjuvant treatment.

## Introduction

Overall, roughly one-third of non-small cell lung cancers (NSCLC) have a mutation in the epidermal growth factor receptor (*EGFR*) gene, although distinct geographical differences have been reported, with the lowest in Europe and the highest in Asia (1). The majority of mutations in *EGFR* comprise deletion of exon 19 and point mutations consisting of L858R, residing in Exon 21 (2, 3), with the remainder being uncommon mutations (4, 5). The increasing use of next-generation sequencing (NGS) and other methods in routine practice has led to an enhanced ability to detect both common and rare variants (6). Improved knowledge of the mutational spectrum of *EGFR* in NSCLC has also allowed for better targeting of the available tyrosine kinase inhibitors to the individual patient (6).

EGFR-tyrosine kinase inhibitors (EGFR-TKIs) are among the front-line agents used for *EGFR*-mutant NSCLC, and several first-, second-, and third-generation EGFR-TKIs have been developed, starting from gefitinib (7, 8). Compared with first-generation EGFR-TKIs, second- and third generation EGFR-TKIs were demonstrated to add clinical benefits in this setting Significant improvement in overall survival (OS) was seen with osimertinib in the FLAURA trial (9–11).

Osimertinib is a 3rd-generation EGFR-TKI that is associated with lasting response in patients with NSCLC harboring the most frequent *EGFR* mutations (10, 12).

Based on the initial results from the Phase 3 ADAURA trial, osimertinib was approved for the treatment of resectable *EGFR*-mutated NSCLC after surgical removal of the tumor. In ADAURA, 90% of patients with stage II-IIIA disease receiving osimertinib were alive and free of cancer at 2 years, compared with 44% of those receiving placebo (13). The ADAURA study is still ongoing and the definitive results, including overall survival, are expected to be released in the future. Osimertinib is also being studied in the Phase III NeoADAURA trial (NCT04351555), which is assessing neoadjuvant osimertinib with or without chemotherapy versus chemotherapy alone prior to surgery, in patients with resectable stage II-IIIB N2 *EGFR* mutation-positive NSCLC (14).

At present, in daily practice, real-world approaches to NSCLC differ greatly, especially in terms of testing frequency of *EGFR* that can be used to drive clinical decisions (15, 16). In Italy, reflex *EGFR* molecular assessment in surgical samples from resected patients is not standard practice and this may have a significant impact on the treatment pathway and outcomes.

The aim of the present study was to characterize the real-world diagnostic-therapeutic pathway and clinical outcomes of early-stage NSCLC, with particular reference to the *EGFR*-mutant subgroup, at a reference thoracic oncology unit in the Veneto Region. A final evaluation of costs for cancer care in this setting was also carried out.

## Materials and Methods

### Study Design and Patients

This is an observational study including a retrospective series of patients with resected non-squamous NSCLC patients referred, between January 2017 and March 2019, to the Thoracic Surgery Unit of the Department of Cardio-Thoracic and Vascular Sciences, University Hospital of Padova and to the Medical Oncology Unit of the Comprehensive Cancer Center Veneto Institute of Oncology (IOV). Main inclusion criteria were: age > 18 years, histologically confirmed diagnosis of NSCLC, chemo-naïve early stage or recurrent disease patients (stage I-IIIB according to 8th edition of the TNM Classification of Malignant Tumors) who underwent radical surgical resection. The study was conducted in accordance with Good Clinical Practice guidelines and the Declaration of Helsinki. The study was approved by the IOV Ethical Committee and all patients signed a specific Informed Consent Form, according to Regulation (EU) 2016/679 of the European Parliament and the Council on personal data protection.

Clinical characteristics included age, gender, residence, smoking status, occupational exposure, symptoms at diagnosis, and family history for neoplasms. Pathological data included histological tumor pattern, vascular and perineural invasion, spread through air spaces (STAS), fibrosis, necrosis, inflammation score, number of mitoses, proliferative index (Ki-67), mucinous secretion, pathological disease stage, and molecular profile.

The diagnostic-therapeutic pathway was tracked, registered, and measured using specific indicators (**Supplementary Table 1**). The process of biopsy sampling, if present, along with specimen handling and pathological procedures for histological classification and molecular assessment were collected. Moreover, we collected systemic and locoregional treatment approaches in the adjuvant setting. At disease relapse, we recorded the site of relapse, further treatments, and relative response, when available. Finally, patient status and date of death or last follow-up was recorded.

In addition, administrative data from a subset of patients with a diagnosis of early-stage NSCLC, were extracted in order to track diagnostic-therapeutic procedures and costs of the overall path.

### Endpoints

The main objective of the study was to describe the diagnostic-therapeutic pathway of non-squamous NSCLC patients who underwent radical surgical resection through specific indicators (**Supplementary Table 1**). In particular, our primary objective was to evaluate the following data: a) the proportion of *EGFR* mutation analyses performed autonomously by the pathologist (reflex test); b) turnaround time (TAT) expressed in calendar days between the date of biopsy/specimen acceptance at the pathology unit and histologic report (including *EGFR* mutation test); c) systemic treatment at the time of disease relapse. The secondary objectives of the study were to evaluate, in a real-world setting, survival and treatment outcomes in the overall population and in patients with mutated *EGFR*, in terms of: a) median relapse-free survival (mRFS), measured as the time from surgery to first evidence of disease relapse; b) median overall survival (mOS), measured as the time between surgery and death for any cause. A description of treatment received at relapse was also reported. An exploratory objective was to assess the influence of clinical-pathological features on outcomes in order to identify independent predictive and prognostic factors.

Finally, a cost analysis for the management of NSCLC patients undergoing radical surgery, including molecular analysis, was also performed in a subset of patients.

### Molecular Testing

*EGFR* mutations in exons 18-21 were tested at diagnosis on tumor biopsy/cell blocks or on tumor specimens. EGFR rare/uncommon mutations are defined as alterations with the exception of common sensitizing exon 19 deletion, L858R point mutation and T790M mutation, accounting for about 15% to 20% of all EGFR mutations. Complex or compound mutations are usually defined as the presence of two or more different EGFR mutations in the same tumor sample. For analyses of tissue samples, tumor DNA was extracted from formalin-fixed paraffin-embedded (FFPE) tumor slices using the QIAamp DNA FFPE kit (Qiagen, Hilden, Germany), and quantified with a Nanodrop spectrophotometer (NanoVue, GE Healthcare, Milwaukee, WI, USA); DNA sequencing was carried out with Sanger sequencing, pyrosequencing, polymerase chain reaction (PCR)-based methods (easy *EGFR* kit, Diatech Pharmacogenetics, Jesi, Italy; *EGFR* mutation analysis kit EntroGen, EntroGen, USA), and mass spectrometry-based methods (Myriapod lung status kit, Sequenom MassARRAY, Diatech Pharmacogenetics, Jesi, Italy) (17).

### Cost Analysis

The cost analysis was performed on anonymized aggregate data from patients referring to our center in 2017. Data on drug prescriptions, use of medical devices, hospital admissions, visits to outpatient clinics and the emergency room, and hospice admissions were drawn from the administrative databases. In particular, costs were drawn from the reimbursement rates established by the Veneto Regional Authority for each procedure or medical action, as previously described (17). We assessed overall costs within 2 years of follow-up after NSCLC was diagnosed. The average per-patient, stage-specific, real-world costs were calculated. Moreover, methods for *EGFR* mutation detection adopted at the referral center within the time-frame of interest were collected and costs of each analysis were calculated on a per-patient basis according to reimbursement rates established by the Veneto Regional Authority.

### Statistical Analysis

All statistical analyses were performed with SPSS Statistics for Windows, Version 26.0 (IBM Corp., Armonk, NY), mRFS and mOS were estimated using the Kaplan-Meier method, and the log-rank test was used to compare survival between groups. Hazard ratios (HRs) and 95% CI were calculated with the Cox proportional hazard model; these analyses were applied to identify the impact of each clinical-pathological feature on outcome as mentioned above. Statistical significance was set at p<0.05.

## Results

### Patient Characteristics, Radical Treatment, and Pathological Features

The characteristics of the study population are detailed in **Table 1**. The cohort included 225 patients, all of whom had adenocarcinoma, with a median age of 70 years of whom 57% were male. Among those screened (223) 44 patients (20%) had adenocarcinoma harboring *EGFR* mutations (**Table 1**). Most patients had stage I at diagnosis (IA, 20%; IB, 39%). Lobectomy was the most common surgical procedure used and was performed in 163 patients (72%). Pathological features are listed in **Table 2**. Most cases *(N=*155, 69%*)* had prevalent acinar histology and a combination of lepidic/acinar and acinar/acinar pattern *(N=*217, 96.4%). Nodal, vascular, perineural, alveolar, and pleural invasion was present in 49 (22%), 99 (44%). 13 (6%), 117 (52%), and 145 cases (64%), respectively.

**Table 1:** Patient characteristics.

Variable	*N* (%)
Number of cases	225
Age (years), median (range)	70 (45-89)
Gender	
Male	129 (57)
Female	96 (43)
Smoking status	
Never smokers	42 (18)
Former smokers	92 (41)
Smokers	69 (31)
Unknown	22 (10)
Occupational Exposure	
Yes	32 (14)
No	164 (73)
Unknown	29 (13)
Molecular status	
EGFR	44 (20)
Ex 19	24 (11)
Ex 21	10 (4)
Rare	5 (2)
Complex	5 (2)
ALK	9 (4)
ROS	7 (3)
KRAS	19 (9)
BRAF	3 (1)
HER2	0 (0)
No mutations	141 (63)
Stage at diagnosis	
I	
IA	45 (20)
IB	87 (39)
II	
IIA	13 (6)
IIB	34 (15)
IIIA	32 (14)
IIIB	10 (4)
NA	4 (2)
Type of surgery	
Bilobectomy	10 (4)
Lobectomy	163 (72)
Sleeve lobectomy	2 (1)
Pneumectomy	8 (4)
Segmentectomy/typical resection	14 (6)
Atypical resection	13 (6)
Other	15 (7)
Number of nodal stations removed, median (range)	8 (0-15)
Adjuvant systemic therapy	
Yes	41 (18)
No	174 (77)
Unknown	10 (4)
Adjuvant radiotherapy	
Yes	14 (6)
No	201 (89)
Unknown	10 (4)

NA, not applicable; Ex, exon.

**Table 2:** Pathological features.

Variable	*N* (%) N = 225	
Prevalent growth pattern		
Lepidic	18	(8)
Acinar	155	(69)
Papillary	8	(4)
Solid	41	(18)
NE	3	(1)
Proliferative index (Ki-67)		
<20%	69	(30)
≥20%	127	(56)
NE	29	(13)
TIL Score		
≤ 30	190	(84)
>30	34	(15)
NE	1	(0)
Tumor necrosis		
≤30	191	(85)
>30	27	(12)
NE	7	(3)
Combination of patterns		
Lepidic/acinar	132	(59)
Acinar/acinar	92	(41)
NE	1	(0)
Nodal invasion		
Yes	49	(22)
No	168	(74)
NA	8	(4)
Fibrosis score		
≤30%	168	(75)
>30%	21	(9)
NE	36	(16)
Vascular invasion		
Yes	99	(44)
No	125	(56)
NE	1	(0)
Perineural invasion		
Yes	13	(6)
No	211	(94)
NE	1	(0)
STAS		
Yes	117	(52)
No	107	(48)
NE	1	(0)
Pleural infiltration		
Absent	73	(32)
Present	145	(64)
NE	7	(3)
Mucinous secretion		
Yes	47	(21)
No	177	(79)
NE	1	(0)
Number of mitoses		
<5/10 HPF	156	(69)
≥5/10 HPF	28	(12)
NE	41	(18)

NE, not evaluated; TIL, tumor infiltrating lymphocyte; NA, not applicable; HPF, high-power field; STAS: spread through air spaces.

### Diagnostic-Therapeutic Pathway

**Figure 1:**
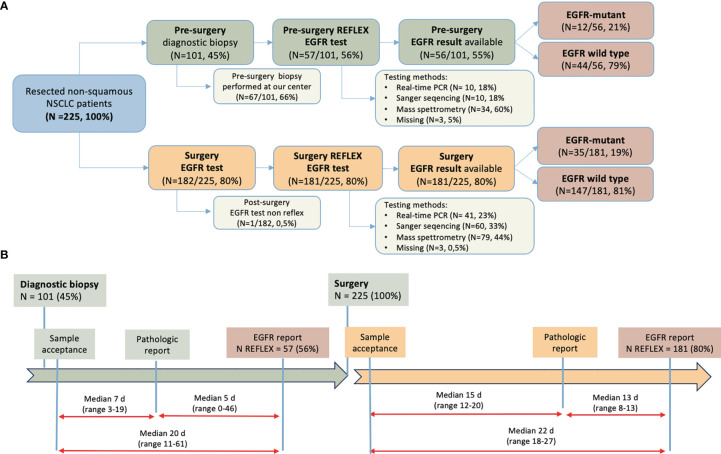
Diagnostic-therapeutic pathway of patients enrolled. **(A)** Patient flow according to EGFR-testing time, material, methods used, and result; **(B)** Turnaround time between diagnostic procedure and pathological and molecular report, pre- and post-surgery. NSCLC, non-small-cell lung cancer; EGFR, epidermal growth factor receptor; N, number; PCR, polymerase chain reaction; d, days.

Details on the diagnostic procedures are depicted in **Figure 1**. No patient received neoadjuvant systemic treatment.

Prior to surgery, 101 patients had undergone diagnostic biopsy; 67 of the 101 (66%) patients with diagnostic biopsy had undergone the procedure at the same center in Padua. Prior to surgery, *EGFR* testing was performed on 57 patients (56%) who had undergone diagnostic biopsy, although *EGFR* mutational results were only available in 56 (55%) patients because of one case of failed analysis. An *EGFR* mutation was found in 12 cases (21%). Among those analyzed, a mass spectrometry-based method was most frequently used method (34 of 57, 60%). After surgery, a reflex EGFR test was performed in 181 (80%) of 225 cases. Overall, a reflex *EGFR* test (pre- or post-surgery) was performed in almost all samples (221 of 225; 98%) with the surgical specimen used in most cases (74%). Of note, in 14 cases an *EGFR* test was performed both pre- and post-surgery. Among surgical specimens, *EGFR* was mutated in 35 cases (19%) and was wild-type in 147 cases (81%).

The median TAT for key pathological analyses is shown in **Figure 1B**. The median time from pre-surgical biopsy sample registration to pathological report was 7 calendar days (range 3-19 days), and from pathological report to *EGFR* report an additional 5 days (range 0-46). Following surgery, the definitive pathological report was available after a median of 15 days (range 12-20), and another median of 13 days (range 8-13) was needed for the *EGFR* report. Median time from surgery to *EGFR* report was 22 calendar days (range 18-27).

### Post-Surgery Treatments

Median follow-up from the date of surgery to the last follow-up or death was 36 months. Adjuvant chemotherapy or adjuvant radiotherapy was administered in 51(23%) and 24(11%) patients, respectively (**Table 1**). Treatments administered at disease relapse are reported in **Supplementary Table 2**. Of 49 patients experiencing a disease relapse (21.7% of all population), 6 (12.2%) patients had an *EGFR* mutant adenocarcinoma and 43 (87.8%) were EGFR wild type.

Twenty-six (53%) received a first-line systemic treatment: 8 (31%) chemotherapy, 7 (27%) a single-agent immune checkpoint inhibitor, 7 (27%) a tyrosine kinase inhibitor (TKI) and 2 (8%) chemo-immunotherapy. In two cases (7.7%), the type of treatment was unknown. All *EGFR*-mutant relapsed patients (*N*=6, 12.2%) received an EGFR-TKI: 3 (6.1%) osimertinib, one (2%) gefitinib, one (2%) afatinib. In one *EGFR*-mutant relapsed patient, the specific drug was unknown. One (2%) patient received a KRAS TKI (sotorasib) as first line treatment at the time of disease relapse.

At disease relapse, 20 patients (43%) received locoregional treatments: 13 (65%) radiotherapy and 7 (35%) a surgical procedure. 10 patients (20.4%) received only locoregional treatments at relapse. In 12 cases (24.5%), no data on systemic and locoregional treatments was available.

### Survival Analyses

**Figure 2:**
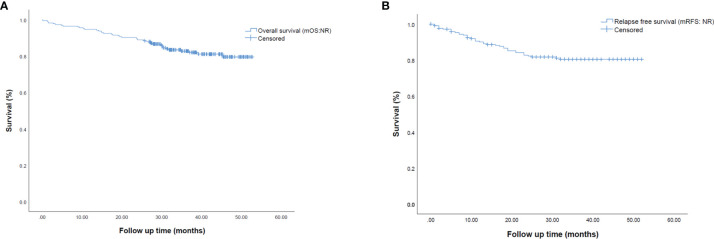
Kaplan-Meier survival curves representing **(A)** median overall survival (mOS) and **(B)** median relapse-free survival (mRFS) in the overall population of early-stage resected NSCLC patients.

**Figure 3:**
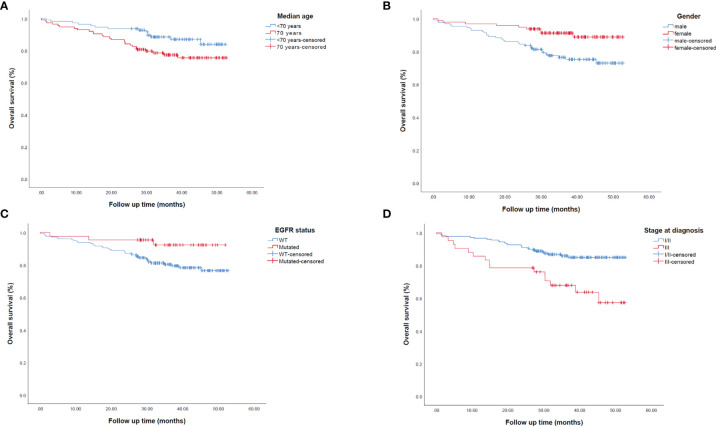
Kaplan-Meier survival curves representing median overall survival (mOS) in early-stage resected NSCLC patients according to **(A)** median age, **(B)** gender, **(C)** *EGFR* status and **(D)** stage at diagnosis. EGFR, epidermal growth factor receptor; WT, wild-type.

Regarding survival outcomes, mOS and mRFS for the entire population were not reached (**Figure 2**). Among variables explored in univariate analysis (**Supplementary Table 3**), OS was significantly longer in patients with age < 70 years (p=0.040) (**Figure 3A**), females (p=0.006) (**Figure 3B**), patients harboring an EGFR mutation (p= 0.044) (**Figure 3C**), not undergoing pneumonectomy (p=0.001), without a solid growth pattern (p=0.008), necrosis score ≤30% (p<0.0001), a combination of lepidic/acinar and acinar/acinar patterns (p=0.003), stage I-II (p=0.001) (**Figure 3C**), no nodal involvement (p=0.006), no vascular invasion (p=0.04), no perineural invasion (p<0.0001), and without relapse (p<0.0001). In particular, two-year survival was 100% in stage I, and decreased to 90% and 64% for stages II and III, respectively.

Multivariate analysis for OS confirmed the correlation with age (p=0.035; HR 1.052, 95% CI 1.004-1.104), female gender (p=0.008, HR 0.032; 95% CI 0.125-0.727), EGFR status (p=0.032, HR 0.190; 95% CI 0.042-0.866), necrosis score (p=0.12, HR 3.299, 95% CI, 1.294-8.414), perineural invasion (p=0.034, HR 2.989, 95% CI, 1.086-8.222), and relapsed disease (p=0.004, HR 2.856, 95% CI 1.396-5.841) (**Supplementary Table 3**). As far as the impact on *EGFR* mutation on survival is concerned, univariate and multivariate analyses showed its predictive role and impact of the targeted treatment at the time of disease relapse on survival.

**Figure 4:**
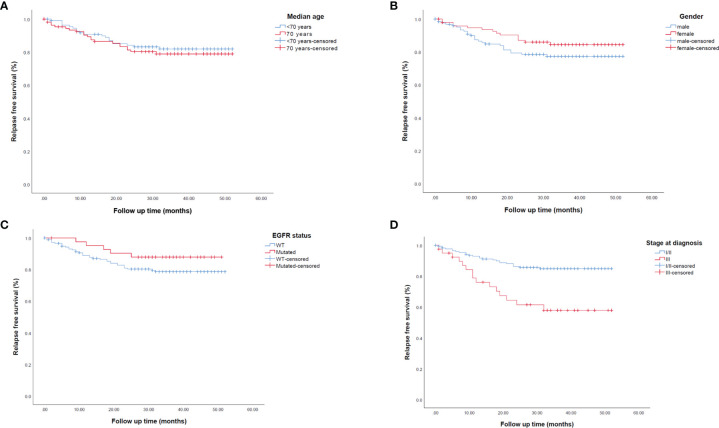
Kaplan-Meier survival curves representing median relapse-free survival (mRFS) in early-stage resected NSCLC patients according to **(A)** median age, **(B)** gender, **(C)** *EGFR* status and **(D)** stage at diagnosis. EGFR, epidermal growth factor receptor; WT, wild-type.

Univariate and multivariate analyses for mRFS are reported in **Supplementary Table 4**. We found significantly longer mRFS in patients without a solid growth pattern (p=0.007), necrosis score ≤30% (p<0.0001), a combination of lepidic/acinar and acinar/acinar patterns (p=0.019), stage I or II (p<0.0001) (**Figure 4D**), and no vascular invasion (p=0.017). No difference in mRFS was seen for age, gender, or *EGFR* status. (**Figures 4A–C**). Multivariate analysis for mRFS (**Supplementary Table 2**) confirmed the correlation within necrosis score (p=0.001; HR 4.579, 95% CI 1.927-10.880), stage at diagnosis (p=0.047, HR 2.272, 95% CI 1.012-5.102), and occupational exposure (p=0.013, HR 2.696, 95% CI, 1.232-5.901).

### Cost Analysis

A cost analysis from administrative data flows showed average costs of € 34,340.40 (range 28,423.20 - 40,257.50) for the management of 2017 early NSCLC cases undergoing radical surgery (2 years after diagnosis), with a 2-year survival of 79.6%. When overall costs were subdivided according to stage, we observed a medium cost of €25,784.20 (16,016.44 - 35,551.96) in stage I; €29,842.20 (22,033.91 - 37,650.49) in stage II; €39,608.65 (29,243.86 - 49,973.44) in stage III. Mean costs of real time (RT)-PCR and Sanger sequencing was €544.55 and €814.70 per patient, respectively, according to reimbursement rates established by the Veneto Regional Authority.

## Discussion

The present real-world analysis in patients with early-stage NSCLC evaluated baseline characteristics, diagnostic and treatment patterns, and TAT of pathological analyses, as well as outcomes and costs. Median age is in line with previously published observational studies (18, 19). While one-fifth of patients had mutations in *EGFR*, over 60% had no detectable mutations. Lobectomy was performed in the majority of patients.

At disease relapse, almost a quarter of patients received only locoregional treatment and all *EGFR*-mutant patients received an EGFR-TKI (20, 21), as described in previous similar series of EGFR mutant NSCLC, eventually followed by a variety of combination therapies (22, 23).

Tissue rebiopsy at the time of disease relapse after radical surgery was not the standard of care to drive treatment decisions, considering that this procedure is mostly invasive and sometimes not feasible. Currently, the regional document about the diagnostic-therapeutic pathway of lung cancer patients, recommend tissue rebiopsy in this setting after multidisciplinary team discussion and consideration of the time interval between the primary treatment and disease relapse. A prospective study aiming at evaluating the concordance of the molecular status between diagnostic biopsy, surgical specimen and relapsed biopsy, should be warranted.

In the overall population, median OS was not reached, with small differences observed in different subgroups of patients. Most real-world studies have been performed on patients with advanced NSCLC, and thus comparison of OS in early-stage tumors in the present study is difficult. For example, in the study by Arriola et al. in advanced *EGFR*-positive NSCLC, OS was 21.4 months in those with exon 19 deletions and 11.1 months in those with rare mutations (22).

Regarding the diagnostic-therapeutic pathway, *EGFR*-reflex test was performed on virtually all surgical samples using a variety of methods; it is of interest that *EGFR* status was available in only 55% of patients undergoing biopsy prior to surgery and that in 14 cases (6%) the test was repeated post-surgery. The repetition of molecular analyses on surgery specimen is regulated in our center according to a specific standard operation procedure (SOP) to improve allocation of resources and avoid unneeded tests (e.g. the molecular analysis is repeated when the percentage of neoplastic cells is <20%).

As far as the timing of diagnostic procedures is concerned, TAT from pathologic report of biopsy samples to *EGFR* report (5 days) is in line with another real-world study from our group, including *EGFR*-mutant advanced NSCLC patients (24). In contrast, TAT from post-surgery pathological reported for the *EGFR* test was longer (13 days). This could be due to the different molecular testing methods that are preferably used. Indeed, in a subset of surgical specimens (cases of 2017 and many of 2018) Sanger sequencing was more frequently used than RT-PCR. Sanger sequencing can be used in cases with a higher number of neoplastic cells as is usually the case in surgical specimens, but is labor intensive and time consuming.

The importance of testing NSCLC for *EGFR* mutations at earlier stages of management is further reinforced by the recent results of the ADAURA trial which enrolled patients with NSCLC harboring *EGFR* exon 19 deletions or exon 21 L858R mutations (13). Early testing would allow for earlier identification of patients eligible to adjuvant osimertinib. Recent results from CTONG1104 have shown that adjuvant treatment with gefitinib is associated with superior disease-free survival, reduced toxicity, and improved quality of life, although the benefit did not translate into an advantage in OS (25). The importance of testing should also be considered in light of the ongoing NEOADAURA trial (NCT04351555) (14), where patient selection on the presence of *EGFR* mutation is required. Indeed, the option of a targeted treatment in the adjuvant setting may have an impact on a sensitive detection of disease relapse by scheduling monthly clinical control visits in patients under treatment. This could imply a new characterization of the tumor at the time of disease relapse by using tissue re-biopsy or liquid biopsy to tailor treatment with new targeted drugs.

Finally, we reported a cost analysis assessed in the real-world. Mean costs of the management of early NSCLC patients progressively increased from stage I to stage III due to greater need of medical assistance. Depending on the method used, *EGFR* test costs represented 1.6-2.4% of the total cost of management per patient (€34,340.40). In the ADAURA trial, an 83% reduction in risk of recurrence or death with adjuvant osimertinib in stage II/IIIA disease (HR: 0.17; P*<*0.0001) was reported (13). More recently, Buja and Pasello showed a reduction in 2-year survival for patients in stage II from 2015 to 2017 in the Veneto Region (84.6% in 2015 compared with 83.3% in 2017), underling the need to improve treatment in this setting (26).

One strength of the present study is the comprehensive analysis of the diagnostic and treatment pathway carried out in all stages of resectable NSCLC. This study however has some limitations. First, this is a single-center retrospective design, although it does include patients who underwent surgery in the Thoracic Surgery Unit at the Department of Cardio-Thoracic and Vascular Sciences of the University of Padua, a referral center also for patients outside the Veneto Region. This allowed a standardized treatment and a uniform collection of clinical data.

Second, the study recruited a relatively limited number of patients. Nevertheless, very few data are reported in the literature on the real-world diagnostic pathway including cost-consequences of *EGFR* testing in early-stage NSCLC patients (27). This data would be useful for different stakeholders (clinician, patients, policymakers, and payers) to assess the benefit and sustainability of interventions in daily clinical practice.

The real-world diagnostic-therapeutic pathway of early-stage NSCLC demonstrated a similar occurrence of *EGFR* mutations to advanced tumors. Reflex *EGFR* testing in all early-stage NSCLC at diagnosis or after surgery may be a sustainable approach to give patients the best chance to benefit from targeted adjuvant treatment and prevent disease relapse.

## Data Availability Statement

The original contributions presented in the study are included in the article/**Supplementary Material**. Further inquiries can be directed to the corresponding author.

## Ethics Statement

The studies involving human participants were reviewed and approved by IOV Ethical Committee. The patients/participants provided their written informed consent to participate in this study.

## Author Contributions

GPa: Conceptualization; Data curation; Formal analysis; Funding acquisition; Investigation; Methodology; Project administration; Supervision; Validation, Writing - original draft; Writing - review and editing. ML and GPr: Data curation; Formal analysis; Methodology; Writing - original draft GC, FP, MS, SF, and LB: Data curation, Investigation, Validation; AB: Data curation; Formal analysis; Methodology; Writing - review and editing. FC, VG, and FR: Conceptualization, Supervision; Validation, Writing review and editing. All authors contributed to the article and approved the submitted version.

## Funding

Support from AstraZeneca for development of this manuscript is acknowledged. The funder was not involved in the study design, collection, analysis, interpretation of data, the writing of this article or the decision to submit it for publication.

## Conflict of Interest

GPa: Advisory Boards/Honoraria/Speakers’ fee/Consultant for: AstraZeneca, BMS, Boehringer Ing., Eli Lilly; MSD, Novartis, Pfizer, Roche, Takeda; VG: personal fees from Eli Lilly, Roche, Novartis, MSD, GSK, Gilead, outside the submitted work.

The remaining authors declare that the research was conducted in the absence of any commercial or financial relationships that could be construed as a potential conflict of interest.

## Publisher’s Note

All claims expressed in this article are solely those of the authors and do not necessarily represent those of their affiliated organizations, or those of the publisher, the editors and the reviewers. Any product that may be evaluated in this article, or claim that may be made by its manufacturer, is not guaranteed or endorsed by the publisher.
