# Supplementary material for: Diagnostic-Therapeutic Pathway and Outcomes of Early Stage NSCLC: a Focus on EGFR Testing in the Real-World
Source: Front Oncol. 2022 Jun 29;12:909064. doi: 10.3389/fonc.2022.909064 (PMC9278847; doi:10.3389/fonc.2022.909064)
Supplement: Supplementary file 1 [file Table_1.docx]

Supplementary Material

**Supplementary Table 1. Indicators for the monitoring of diagnostic-therapeutic pathway of patients involved into the study.**

| **INDICATOR** | **TIME** | **Administrative flow extractable** |
| --- | --- | --- |
| 1. proportion of patient s with PET-CT scan within 60 days before surgery (T2 or greater and/or N1 or greater) | Diagnosis/staging | Yes |
| 2. Waiting time for surgery | Surgical treatment | Yes |
| 3. Proportion of patients referred to multidisciplinary meeting before or after surgery (60 days pre and post) | Diagnostic-therapeutic program | No |
| 4.a Mortality within 30 days after surgery | Surgical treatment | Yes |
| 4.b Mortality within 90 days after surgery | Surgical treatment | Yes |
| 5. Proportion of patients undergoing pneumectomy | Surgical treatment | Yes |
| 6. Proportion of patients undergone sleeve resection | Surgical treatment | Yes |
| 7. Proportion of patients with negative margins after radical surgery | Surgical treatment | No |
| 8. Proportion of patients receiving surgical sampling in more than 4 node stations | Surgical treatment | No |
| 9. Proportion of advanced NSCLC patients with molecular analysis at diagnosis/relapsed | Medical treatment | No |
| 10. Proportion of advanced NSCLC patients with molecular analysis available within 10 working days from sample shipment to laboratory: molecular reporting time | Medical treatment | No |
| 11. Proportion of EGFR mutation analyses performed autonomously by the pathologist (reflex test) in early-stage NSCLC patients | Surgical treatment | No |
| 12. TAT between the date of biopsy/specimen’s reception at the pathology unit and histologic report (including EGFR mutation test). | Surgical treatment | No |

Abbreviations: NSCLC, non-small-cell lung cancer; EGFR, epidermal growth factor receptor; PET-CT, positron emission tomography-computed tomography; TAT, turnaround time

**Supplementary Table 2. Treatments administered at relapse.**

| **Variable** | | | | ***N* (%)**  **N=49** | |
| --- | --- | --- | --- | --- | --- |
|  |  |  |  |  |  |
| **First line treatment** | | | |  |  |
|  | Yes | | | 26 | (53) |
|  | **Type of first line treatment** | | |  |  |
|  |  | | Tyrosine kinase inhibitors | 7 | (27) |
|  |  | | Immunotherapy | 7 | (27) |
|  |  | | Chemotherapy | 8 | (31) |
|  |  | | Chemo-immunotherapy | 2 | (8) |
|  |  | | Unknown | 2 | (8) |
|  | No | | | 16 | (32) |
|  | Unknown | | | 7 | (14) |
| **Locoregional treatments** | | | |  |  |
|  | Yes | | | 19 | (39) |
|  |  | Radiotherapy | | 12 | (63) |
|  |  | Surgery | | 7 | (37) |
|  | No | | | 19 | (39) |
|  | Unknown | | | 11 | (22) |

Abbreviation: N, number.

**Supplementary Table 3. Univariate and multivariate analysis for overall survival**

|  |  | | **OS univariate analysis** | | | **OS multivariate analysis** | |
| --- | --- | --- | --- | --- | --- | --- | --- |
| **Variables** | ***N (%)*** | | **mOS, months (95% CI)** | | **p value** | **p value** | **HR (95% CI)** |
| **Age (years)** |  | |  | |  |  |  |
| <70 | 111 (49) | | NR | | .*040* | *.035* | 1.052 (1.004-1.104) |
| ≥70 | 114 (51) | | NR | |  |  |  |
| **Gender** |  | |  | |  |  |  |
| Male | 129 (57) | | NR | | *.006* | *.008* | 0.302 (0.125-0.727) |
| Female | 96 (43) | | NR | |  |  |  |
| **Smoking Status** |  | |  | |  |  |  |
| Smokers | 161 (72) | | NR | | .416 | - | - |
| Never smokers | 42 (18) | | NR | |  |  |  |
| Unknown | 22 (10) | | - | |  |  |  |
| **EGFR status** |  | |  | |  |  |  |
| WT | 181 (80) | | NR | | *.044* | *.032* | 0.190 (0.042-0.866) |
| Mutant | 44 (20) | | NR | |  |  |  |
| **Type of surgery** | |  | |  |  |  |  |
| Pneumonectomy | | 8 (4) | | 30.3 (25.433-35.167) | *.001* | .558 | 1.453 (0.417-5.070) |
| Other | | 217 (96) | | NR |  |  |  |
| **Prevalent growth pattern** |  | |  | |  |  |  |
| Solid | 41 (18) | | NR | | *.008* | .474 | 0.705 (0.271-1.836) |
| Other | 181 (80) | | NR | |  |  |  |
| NE | 3 (1) | | - | |  |  |  |
| **Proliferative index (Ki67)** |  | |  | |  |  |  |
| <20% | 69 (30) | | NR | | .851 | - | - |
| ≥20% | 127 (56) | | NR | |  |  |  |
| NE | 29 (13) | | - | |  |  |  |
| **TIL** |  | |  | |  |  |  |
| ≤30 | 190 (85) | | NR | | .392 | - | - |
| >30 | 34 (15) | | NR | |  |  |  |
| NE | 1 (0) | | - | |  |  |  |
| **Tumor necrosis** |  | |  | |  |  |  |
| ≤30% | 191 (85) | | NR | | *<.0001* | *.012* | 3.299 (1.294-8.414) |
| >30% | 27 (12) | | 30.330 (27.562-33.098) | |  |  |  |
| Unknown | 7 (3) | | - | |  |  |  |
| **Combination of patterns** |  | |  | |  |  |  |
| Lepidic/acinar | 132 (59) | | NR | | *.003* | .256 | 1.662 (0.692-3.992) |
| Acinar/acinar | 92 (41) | | NR | |  |  |  |
| NE | 1 (0) | | - | |  |  |  |
| **Stage at diagnosis** |  | |  | |  |  |  |
| I/II | 179 (80) | | NR | | *.001* | .405 | 0.623 (0.205-1.896) |
| III | 42 (18) | | NR | |  |  |  |
| NA | 4 (2) | | - | |  |  |  |
| **Nodal Invasion** |  | |  | |  |  |  |
| Yes | 49 (22) | | NR | | *.006* | .216 | 1.938 (0.680-5.523) |
| No | 168 (74) | | NR | |  |  |  |
| NA | 8 (4) | | - | |  |  |  |
| **Fibrosis Score** |  | |  | |  |  |  |
| ≤30% | 168 (75) | | NR | | .729 | - | - |
| >30% | 21 (9) | | NR | |  |  |  |
| NE | 36 (16) | | - | |  |  |  |
| **Vascular Invasion** |  | |  | |  |  |  |
| Yes | 99 (44) | | NR | | *.040* | .596 | 1.246 (0.552-2.810) |
| No | 125 (56) | | NR | |  |  |  |
| NE | 1 (0) | | - | |  |  |  |
| **Perineural Invasion** |  | |  | |  |  |  |
| Yes | 13 (6) | | NR | | *<.0001* | *.034* | 2.989 (1.086-8.222) |
| No | 211 (94) | | 27.730 (24.122-31.338) | |  |  |  |
| NE | 1 (0) | | - | |  |  |  |
| **STAS** |  | |  | |  |  |  |
| Yes | 117 (52) | | NR | | .703 | - | - |
| No | 107 (48) | | NR | |  |  |  |
| NE | 1 (0) | | - | |  |  |  |
| **Pleural Infiltration** |  | |  | |  |  |  |
| Absent | 73 (32) | | NR | | .664 | - | - |
| Present | 145 (64) | | NR | |  |  |  |
| NE | 7 (3) | | - | |  |  |  |
| **Mucinous secretion** |  | |  | |  |  |  |
| Yes | 47 (21) | | NR | | .146 | - | - |
| No | 177 (79) | | NR | |  |  |  |
| NE | 1 (0) | | - | |  |  |  |
| **Number of mitoses** |  | |  | |  |  |  |
| <5/10 HPF | 156 (69) | | NR | | .080 | - | - |
| ≥5/10 HPF | 28 (12) | | NR | |  |  |  |
| NE | 41 (18) | | - | |  |  |  |
| **Number of nodal stations removed** |  | |  | |  |  |  |
| <8 | 110 (49) | | NR | | .626 | - | - |
| ≥8 | 114 (51) | | NR | |  |  |  |
| Unknown | 1 (0) | | - | |  |  |  |
| **Reflex EGFR test** |  | |  | |  |  |  |
| Yes | 221 (98) | | NR | | .631 | - | - |
| No | 4 (2) | | NR | |  |  |  |
| **Adjuvant chemotherapy** |  | |  | |  |  |  |
| Yes | 41 (18) | | NR | | .608 | - | - |
| No | 174 (77) | | NR | |  |  |  |
| Unknown | 10 (4) | | - | |  |  |  |
| **Adjuvant radiotherapy** |  | |  | |  |  |  |
| Yes | 14 (6) | | NR | | .498 | - | - |
| No | 201 (89) | | NR | |  |  |  |
| Unknown | 10 (4) | | - | |  |  |  |
| **PET <60 days before surgery** |  | |  | |  |  |  |
| Yes | 92 (41) | | NR | | .611 | - | - |
| No | 116 (52) | | NR | |  |  |  |
| Unknown | 17 (7) | | - | |  |  |  |
| **Time from visit to surgery** |  | |  | |  |  |  |
| <64 days | 94 (42) | | NR | | .694 | - | - |
| ≥64 days | 95 (42) | | NR | |  |  |  |
| Unknown | 36 (16) | | - | |  |  |  |
| **Occupational exposure** |  | |  | |  |  |  |
| Yes | 32 (14) | | NR | | .144 | - | - |
| No | 164 (73) | | NR | |  |  |  |
| Unknown | 29 (13) | | - | |  |  |  |
| **Relapse** |  | |  | |  |  |  |
| Yes | 49 (22) | | NR | | *<.0001* | *.004* | 2.856 (1.396-5.841) |
| No | 179 (80) | | NR | |  |  |  |

Abbreviations: N, number; NR, not reached; OS, overall survival; HR, hazard ratio; CI, confidence interval; EGFR, epidermal growth factor receptor; TIL, tumor infiltrating lymphocyte; HPF, high-power field; PET, positron emission tomography; NE: not evaluated; STAS: spread through air spaces

**Supplementary Table 4. Univariate and multivariate analysis for relapse-free survival**

|  | |  | **mRFS univariate analysis** | | **mRFS multivariate analysis** | |
| --- | --- | --- | --- | --- | --- | --- |
| **Variables** | | ***N (%)*** | **mRFS, months (95% CI)** | **p value** | **p value** | **HR (95% CI)** |
| **Age (years)** | |  |  |  |  |  |
| <70 | | 111 (49) | NR | .571 | - | - |
| ≥70 | | 114 (51) | NR |  |  |  |
| **Gender** | |  |  |  |  |  |
| Male | | 129 (57) | NR | 171 | - | - |
| Female | | 96 (43) | NR |  |  |  |
| **Smoking Status** | |  |  |  |  |  |
| Smokers | | 161 (72) | NR | .579 | - | - |
| Never smokers | | 42 (18) | NR |  |  |  |
| Unknown | | 22 (10) |  |  |  |  |
| **EGFR status** | |  |  |  |  |  |
| WT | | 181 (80) | NR | .190 | - | - |
| Mutated | | 44 (20) | NR |  |  |  |
| **Type of surgery** | |  |  |  |  |  |
| Pneumonectomy | | 8 (4) | NR | .314 | - | - |
| Other | | 217 (96) | NR |  |  |  |
| **Prevalent growth pattern** |  | |  |  |  |  |
| Solid | 41 (18) | | NR | *.007* | .573 | 1.327 (0.495-3.559) |
| Other | 181 (80) | | NR |  |  |  |
| NE | 3 (1) | | - |  |  |  |
| **Proliferative index (Ki67)** |  | |  |  |  |  |
| <20% | 69 (30) | | NR | .314 | - | - |
| ≥20% | 127 (56) | | NR |  |  |  |
| NE | 29 (13) | | - |  |  |  |
| **TIL** |  | |  |  |  |  |
| ≤30% | 190 (85) | | NR | .888 | - | - |
| >30% | 34 (15) | | NR |  |  |  |
| NE | 1 (0) | | - |  |  |  |
| **Tumor necrosis** |  | |  |  |  |  |
| ≤30% | 191 (85) | | NR | *<.0001* | *.001* | 4.579 (1.927-10.880) |
| >30% | 27 (12) | | 23.000 (8.184-37.816) |  |  |  |
| Unknown | 7 (3) | | - |  |  |  |
| **Combination of patterns** |  | |  |  |  |  |
| Lepidic/acinar | 132 (59) | | NR | *.019* | .829 | 1.099 (0.465-2.600) |
| Acinar/acinar | 92 (41) | | NR |  |  |  |
| NE | 1 (0) | |  |  |  |  |
| **Stage at diagnosis** |  | |  |  |  |  |
| I/II | 179 (80) | | NR | *<.0001* | *.047* | 2.272 (1.012-5.102) |
| III | 42 (18) | | NR |  |  |  |
| NA | 4 (2) | |  |  |  |  |
| **Nodal Invasion** |  | |  |  |  |  |
| Yes | 49 (22) | | NR | .064 | - | - |
| No | 168 (74) | | NR |  |  |  |
| NA | 8 (4) | |  |  |  |  |
| **Fibrosis Score** |  | |  |  |  |  |
| ≤30% | 168 (75) | | NR | .402 | - | - |
| >30% | 21 (9) | | NR |  |  |  |
| NE | 36 (16) | |  |  |  |  |
| **Vascular Invasion** |  | |  |  |  |  |
| Yes | 99 (44) | | NR | *.017* | .682 | 1.179 (0.536-2.594) |
| No | 125 (56) | | NR |  |  |  |
| NE | 1 (0) | | - |  |  |  |
| **Perineural Invasion** |  | |  |  |  |  |
| Yes | 13 (6) | | NR | .325 | - | - |
| No | 211 (94) | | NR |  |  |  |
| NE | 1 (0) | | - |  |  |  |
| **STAS** |  | |  |  |  |  |
| Yes | 117 (52) | | NR | .976 | - | - |
| No | 107 (48) | | NR |  |  |  |
| NE | 1 (0) | | - |  |  |  |
| **Pleural Infiltration** |  | |  |  |  |  |
| Absent | 73 (32) | | NR | .508 | - | - |
| Present | 145 (64) | | NR |  |  |  |
| NE | 7 (3) | | - |  |  |  |
| **Mucinous secretion** |  | |  |  |  |  |
| Yes | 47 (21) | | NR | .970 | - | - |
| No | 177 (79) | | NR |  |  |  |
| NE | 1 (0) | | - |  |  |  |
| **Number of mitoses** |  | |  |  |  |  |
| <5/10 HPF | 156 (69) | | NR | .474 | - | - |
| ≥5/10 HPF | 28 (12) | | NR |  |  |  |
| NE | 41 (18) | | - |  |  |  |
| **Number of nodal stations removed** |  | |  |  |  |  |
| <8 | 110 (49) | | NR | .132 | - | - |
| ≥8 | 114 (51) | | NR |  |  |  |
| Unknown | 1 (0) | | - |  |  |  |
| **Reflex EGFR test** |  | |  |  |  |  |
| Yes | 221 (98) | | NR | .407 | - | - |
| No | 4 (2) | | NR |  |  |  |
| **Adjuvant chemotherapy** |  | |  |  |  |  |
| Yes | 41 (18) | | NR | .064 | - | - |
| No | 174 (77) | | NR |  |  |  |
| Unknown | 10 (4) | |  |  |  |  |
| **Adjuvant radiotherapy** |  | |  |  |  |  |
| Yes | 14 (6) | | NR | .842 | - | - |
| No | 201 (89) | | NR |  |  |  |
| Unknown | 10 (4) | | - |  |  |  |
| **PET <60 days before surgery** |  | |  |  |  |  |
| Yes | 92 (41) | | NR | .124 | - | - |
| No | 116 (52) | | NR |  |  |  |
| Unknown | 17 (7) | | - |  |  |  |
| **Time from visit to surgery** |  | |  |  |  |  |
| <64 days | 94 (42) | | NR | .556 | - | - |
| ≥64 days | 95 (42) | | NR |  |  |  |
| Unknown | 36 (16) | | - |  |  |  |
| **Occupational exposure** |  | |  |  |  |  |
| Yes | 32 (14) | | NR | *.010* | *.013* | 2.696 (1.232-5.901) |
| No | 164 (73) | | NR |  |  |  |
| Unknown | 29 (13) | | - |  |  |  |
|  |  | |  |  |  |  |

Abbreviations: N, number; NR, not reached; RFS, relapse-free survival; HR, hazard ratio; CI, confidence interval; EGFR, epidermal growth factor receptor; TIL, tumor infiltrating lymphocyte; HPF, high-power field; PET, positron emission tomography; NE: not evaluated; STAS, spread through air spaces
